# Development of the Self-Assessment Self-Disclosure Questionnaire to Examine the Association between Self-Disclosure and Frailty among Community-Dwelling Older Adults in Japan

**DOI:** 10.3390/geriatrics9030067

**Published:** 2024-05-26

**Authors:** Kazuki Yokoyama, Hikaru Ihira, Yuriko Matsuzaki-Kihara, Atsushi Mizumoto, Hideyuki Tashiro, Kiyotaka Shimada, Kosuke Yama, Ryo Miyajima, Takeshi Sasaki, Naoki Kozuka, Nozomu Ikeda

**Affiliations:** 1 Department of Occupational Therapy, School of Health Sciences, Sapporo Medical University, Sapporo 060-8556, Japan; 2 Department of Physical Therapy, School of Health Sciences, Sapporo Medical University, Sapporo 060-8556, Japan; 3 Department of Rehabilitation, Japan Health Care University, Sapporo 062-0053, Japan; 4 Department of Physical Therapy, Faculty of Human Science, Hokkaido Bunkyo University, Eniwa 061-1449, Japan; 5 Department of Neuropsychiatry, School of Medicine, Sapporo Medical University, Sapporo 060-8543, Japan; 6 N Field Home-Visit Nursing Station Dune Sapporo, Sapporo 003-0808, Japan; 7 Ebetsu City Hospital, Ebetsu 067-8585, Japan; 8 Department of Rehabilitation, Hokkaido Chitose College of Rehabilitation, Chitose 066-0055, Japan

**Keywords:** prevention, frailty, older adult, self-disclosure, mental health

## Abstract

Self-disclosure is the attitude of communicating one’s experiences and condition to others and is an indicator of mental health and an open personality. Frailty, characterized by reduced physical and psychological resistance, predicts the incidence of dependency and mortality. Although low self-disclosure may be associated with frailty, there is no scale to measure older adults’ self-disclosure. This cross-sectional study assessed the validity of a self-assessment self-disclosure questionnaire and examined the association between the content of self-disclosures to friends and acquaintances and frailty among community-dwelling older adults. A total of 237 adults aged ≥65 in Japan were surveyed using a mailed self-administered questionnaire in 2021. The self-disclosure scale consisted of 10 items and showed adequate validity. Participants were classified into a robust group (*n* = 117, women 57.3%) and a frailty group (*n* = 120, women 73.3%) using the Kihon Checklist. After adjusting for covariates, multivariate-adjusted logistic regression models revealed frailty was associated with lower self-disclosure of recent positive events, motivation and strengths (indicating strong points) in life, relationships with family and relatives, experiences of work and social activities, and financial status. The proposed questionnaire must still be further tested in other populations, but our initial results may contribute to preventing frailty and improving mental health among community-dwelling older adults.

## 1. Introduction

Population aging is a global challenge. In December 2023, Japan recorded the highest proportion of older adults in the world [1]. Approximately 29.2% of inhabitants were 65 years and older, and 16.3% were 75 years and older [2]. Life expectancy increased to 81.6 years for men and 87.7 years for women in 2020, setting an unprecedented record for both genders [1]. Consequently, measures to extend “healthy life expectancy”, that is, the maximum period during which people can remain healthy, are urgently required. The World Health Organization has identified preventing frailty and enhancing intrinsic capacity as key points for healthy aging [3]. The Japan Geriatrics Society defines frailty as “a condition in which vulnerability to stress increases as physiological reserves decline in old age, leading to functional disability, dependency, and death” [4].

Frailty is the accumulation of deficits in multiple areas, including physical, psychological (e.g., mental health, cognitive dysfunction), and social problems (e.g., living alone, economic deprivation), which collectively increase the risk of disability [4,5,6]. One meta-analysis showed that women had higher frailty index scores than men in every age group [7]. Frailty can be improved using appropriate interventions and lifestyle modifications [4,8]. Research has demonstrated the effects of exercise training interventions [9] and increased physical activity [10] in improving frailty. Social participation and networks are also effective in combating frailty [11,12,13]. Social frailty, such as poor social relationships and social disengagement, has an impact on the risk of future disability [14]. Some older adults have a greater preference for solitude, which is associated with higher levels of loneliness and lower levels of extraversion [15]. Productivity during solitude and enjoyment of solitude are associated with positive subjective well-being among older adults [16]. Therefore, older adults who prefer solitude and are introverted may experience difficulty in practicing self-disclosure with others and participating in activities focused on frailty prevention and social networking.

Self-disclosure, an indicator of openness in personality, is the attitude of revealing personal information to others [17]. According to the social penetration theory, self-disclosure and the communication of intimate information are more likely to increase when an interpersonal interaction becomes a more intimate relationship [18]. Adequate self-disclosure is important for mental health as it strengthens interpersonal relationships [17], relationship efficacy [19], and positive self-esteem [17,20]. People with higher self-esteem are mentally stable and able to self-disclose without becoming anxious and defensive [17]. Additionally, self-disclosure is negatively associated with a sense of loneliness [21,22]. Those who feel lonely are less likely to self-disclose and may have trouble developing social relationships [22]. One meta-analysis revealed that women disclosed slightly more than men, although the target had a relationship with the discloser (i.e., friend, parent, or spouse) [23]. Significant correlations have been found between the amount of self-disclosure and depressive tendency and low social activity in men, and between the amount of self-disclosure and physical symptoms and anxiety in women [24]. In older age, self-disclosure is important for mental health and may be an effective countermeasure against frailty. However, questionnaires to assess the content of self-disclosure in older adults have not been developed. Further, the association between the content of self-disclosures and frailty, and the gender differences in this association, remain unexplored. By clarifying this relationship, it may be possible to utilize the degree of individual self-disclosure in the prevention of frailty. This study aimed to assess the validity of the self-assessment self-disclosure questionnaire and examine the association between self-disclosure to friends and acquaintances and frailty among community-dwelling older adults in Japan, as well as the gender differences in this association.

## 2. Materials and Methods

### 2.1. Research Design

This study employed a cross-sectional design and conducted a survey using a self-administered questionnaire distributed by mail.

### 2.2. Participants

**Figure 1:**
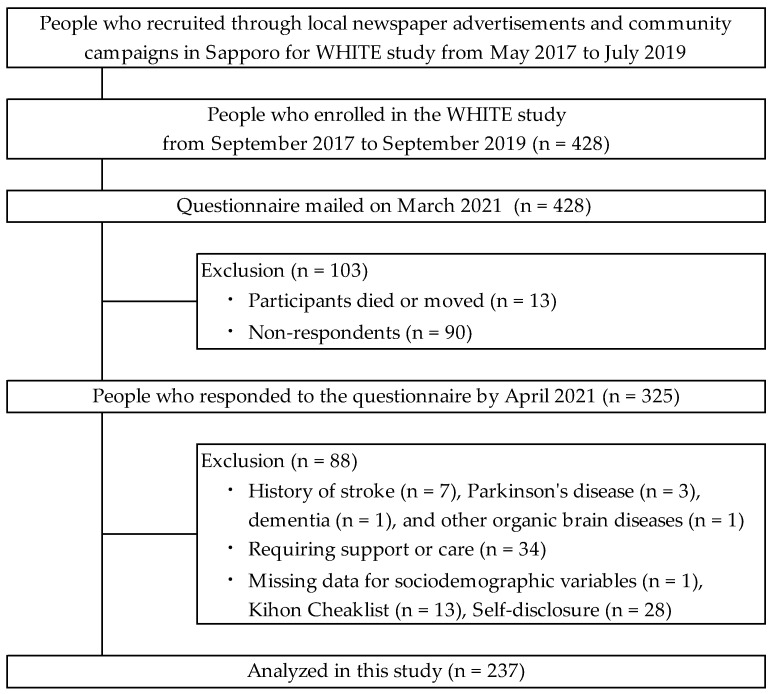
Flowchart of participant inclusion and exclusion.

Figure 1 shows a flowchart of the study participants. The participants were recruited through local newspaper advertisements and community campaigns in Sapporo, Japan for the Widely Hokkaido Individual Training for Elderly (WHITE) study, which conducted a community-based health check survey from May 2017 to July 2019. A total of 428 participants enrolled in the WHITE study from September 2017 to September 2019; a mailed questionnaire survey was conducted in March 2021. Of those mailed, 13 were confirmed to have died or moved, and 90 non-respondents were excluded from the study. There were 325 participants who responded to the present questionnaire by April 2021 (response rate 75.9%). The percentage of women enrolled was 65.4%. The inclusion criteria for participation were individuals aged ≥65 years and those who attended the WHITE study in Sapporo. The exclusion criteria were a history of stroke (*n* = 7), Parkinson’s disease (*n* = 3), dementia (*n* = 1), and other neurological disorders (*n* = 1), and the need for support or care certified by the Japanese public long-term care insurance system (*n* = 34). Participants who provided insufficient sociodemographic data (*n* = 1), or incomplete answers on the Kihon Checklist (KCL; *n* = 13) [25], or self-disclosure (*n* = 28) were excluded. The process, from participant recruitment to data collection and management, was conducted by researchers licensed as occupational or physical therapists affiliated with WHITE. The study was approved by the Sapporo Medical University Ethical Review Board (approval number 28-2-7). Written informed consent was obtained from each participant after the procedure was explained. 

### 2.3. Assessment of Exposure and Outcomes

#### 2.3.1. Frailty Status

Frailty status was assessed using the KCL [25], a self-reported yes/no questionnaire developed by the Japanese Ministry of Health, Labour and Welfare. The KCL comprises 25 items in seven domains: instrumental activities of daily living, physical function, nutritional status, oral function, homebound status, cognitive function, and depressive mood. Each score on the KCL indicates the level of difficulty experienced in performing the activity; a higher score indicates a higher risk of requiring care or support in each domain. A total score of 0–3 points is considered robust, 4–7 points is considered pre-frailty, and ≥8 points is considered frailty according to the Cardiovascular Health Study criteria [25]. The area under the receiver operating characteristic curve for the estimation of pre-frailty status was 0.81 (sensitivity 70.3%, specificity 78.3%), and frailty status was 0.92 (sensitivity 89.5%, specificity 80.7%). The prevalence of frailty and pre-frailty in Japan is reported to be 7.4% and 48.1%, respectively [26]. The total KCL score predicted the incidence of dependency and mortality over three years in community-dwelling older adults [27].

#### 2.3.2. Assessing Self-Disclosure

Self-disclosure was assessed using a 13-item questionnaire. The items were generated with reference to previous studies that have measured self-disclosure in the geriatric population [18,28,29]. In addition, items specific to old age in the context of daily life were extracted from Jourard’s Self-Disclosure Questionnaire [17,30]. As the items in previous questionnaires were created between the 1950s and 1990s [17,18,28,30], we modified them into 13 items adapted to modern times. The amount of self-disclosure across each item was evaluated on a five-point scale (from “1: Hardly ever talk” to “5: Talk often”) using the methods implemented in previous studies with verified reliability [29,31]. Higher scores indicated higher self-disclosure to friends and acquaintances.

#### 2.3.3. Covariates

The covariates were demographic and lifestyle-related variables that impact changes in the association between interpersonal relationships and frailty, including physical, cognitive, psychological, and social domains. The questionnaire collected information on these covariates, including age, gender, years of education, living alone status, work status, and medication status, based on previous studies [7,23,32,33,34,35]. Living alone was identified using the yes/no method. Work status was identified as paid work (full-time or part-time), unpaid work (volunteer), or unemployed. Medication status was recorded as the number of medications taken per day.

### 2.4. Statistical Analyses

For continuous variables, the Kolmogorov–Smirnov tests were conducted. Normal distributions are reported as means and standard deviation (*SD*), while non-normal distributions are reported as median and interquartile range (*IR*). Categorical variables are presented as numbers and percentages (%). Participants were classified into a robust group, or a frailty group (including pre-frailty and frailty) based on their total KCL scores. In the case of normal distributions, the Student’s or Welch’s *t*-test for continuous variables and the *χ*^2^ test for categorical variables were performed to identify differences between the groups.

The validity of the 13 self-disclosure items was examined using data from the study’s participants. It was confirmed that there were no ceiling or floor effects, as the mean ± *SD* was outside the item’s range. In the good-poor (G-P) analysis, we divided the respondents into low- and high-scoring groups and examined differences between groups. Significant differences in average scores for each item were observed between the groups based on the average of the items’ total scores using the *t* test. Correlations between each item’s score and the total score were examined using Spearman’s rank correlation coefficients ≥0.70 in item-total (I-T) correlation analysis. Subsequently, convergent validities were assessed using the correlation between each self-disclosure item and the Japanese version of the World Health Organization-Five Well-Being Index (WHO-5-J) [36]. Thereafter, inter-item correlations were calculated, and any item exceeding 0.7 was removed. 

The items selected through the above process did not indicate normal distributions, so Mann–Whitney U tests were conducted to identify differences between the total KCL scores and each self-disclosure item. Logistic regression analyses were performed with frailty (robust group = 0, frailty group = 1) as the dependent variable and each of the self-disclosure variables as independent variables. In the crude model, the odds ratios (ORs) and 95% confidence intervals (95% CIs) for frailty were calculated according to the self-disclosure content. In adjusted model 1, age and gender were added to the model to examine whether their model associations changed. In adjusted model 2, years of education, medication status, living alone status, and work status were also added to model 1 to examine whether their model associations changed. Additionally, logistic regression analyses were performed to identify gender differences in the association between self-disclosure and frailty, with a self-disclosure and gender interaction term, and *OR*s and 95% CIs were calculated. SPSS version 27.0 (IBM Inc., Armonk, NY, USA) was used for the analyses. The significance level was set at 0.05.

## 3. Results

Among the 237 participants, 117 (49.4%) were assigned to the robust group and 120 (50.6%) to the frailty group. Their mean age was 75.8 (*SD* = 5.1) years. Table 1 summarizes the participants’ demographic characteristics. No significant group differences were observed based on age, years of education, living alone status, or work status. However, the frailty group had a significantly higher proportion of women (*t* (235) = 2.611, *p* = 0.009) and took more medication (*t* (235) = −3.364, *p* < 0.001) than the robust group. For the variables related to frailty in the KCL, the median total score was 2.0 in the robust group and 6.0 in the frailty group (*t* (235) = −21.441, *p* < 0.001). 

Table 2 summarizes the validity of the 13 self-disclosure items. It was confirmed that there were no ceiling or floor effects, as the mean ± *SD* was outside the item’s range. For G-P analysis, the mean scores of all items in the high group were significantly higher than those in the low group. I-T correlation analysis revealed correlation coefficients of all items ≥0.70. Multiple regression analysis, which adjusted for age, gender, years of education, number of medications, living alone status, and work status, revealed that self-disclosure was significantly associated with mental health except for three items related to concerns. Inter-item correlation showed a strong correlation of 0.7 or higher for the following pairs: items 1 and 2, items 3 and 4, items 3 and 6, items 5 and 6, items 6 and 9, and items 12 and 13. Except for three items (concerns about social interaction, physical health, and cognitive function), self-disclosure was significantly associated with mental health. Finally, 10 items were adopted, namely, (a) recent positive events, (b) motivation and strengths in life, (c) personality, (d) future outlook, (e) relationships with family and relatives, (f) experiences of work and social activities, (g) financial status, (h) concerns about social interaction, (i) concerns about physical health, and (j) concerns about cognitive function. 

Table 3 shows group differences in the self-disclosure items. The mean item scores of self-disclosure ranged from 2.3–3.2 and 2.1–3.0 in the robust and frailty groups, respectively. The frailty group had significantly lower scores for motivation and strengths in life (*U* = 5986.5, *p* = 0.041) and experiences of work and social activities (*U* = 5631.0, *p* = 0.006) than the robust group.

Table 4 presents the results of the logistic regression analyses. Compared with the robust group, the frailty group showed significant associations with self-disclosure of recent positive events (*OR* = 0.74; 95% CI: 0.57–0.97, *p* = 0.028), motivation and strengths in life (*OR* = 0.70; 95% CI: 0.53–0.92, *p* = 0.011), relationships with family and relatives (*OR* = 0.75; 95% CI: 0.57–0.99, *p* = 0.042), experiences of work and social activities (*OR* = 0.66; 95% CI: 0.51–0.87, *p* = 0.002), and financial status (*OR* = 0.73; 95% CI: 0.55–0.97, *p* = 0.031), after adjusting for age, gender, years of education, medication status, living alone status, and work status. 

Table 5 shows the results of the logistic regression analyses with the self-disclosure and gender interaction terms. A significant interaction term was observed for self-disclosure of concerns about cognitive function (*OR* = 2.09; 95% CI: 1.19–3.66, *p* = 0.010).

## 4. Discussion

This study aimed to assess the validity of the self-assessment self-disclosure questionnaire and examine the association between self-disclosure to friends and acquaintances and frailty among community-dwelling older adults in Japan. Through a review of previous studies, a 13-item self-disclosure questionnaire was developed. First, the ceiling and floor effects for each item on the self-disclosure questionnaire were investigated and found to be absent. In addition, G-P analyses were performed, which confirmed sufficient internal consistency. Then, focusing on the association between each item of self-disclosure and the WHO-5-J [36], which indicates subjective well-being, convergent validities were tested. Ten items, including items related to daily life and positive aspects of self, were positively associated with the score of WHO-5-J. However, three items, namely, concerns about social interaction, physical health, and cognitive function, showed no significant association. Although several prior studies indicated that an adequate amount of self-disclosure is important for maintaining mental health [17,20,21,22], it was suggested that participants who self-disclose may have more concerns in their daily lives, and that this does not necessarily lead to subjective well-being among community-dwelling older adults. Finally, after confirming the inter-item correlations, six of the patterns exhibited high correlation coefficients, leading to the removal of three items. Through this process, the validity of the self-disclosure questionnaire with 10 items was demonstrated. Ten items of self-disclosure were associated with the total KCL score. Five items of self-disclosure (recent positive events, motivation and strengths in life, relationships with family and relatives, experiences of work and social activities, and financial status) were associated with frailty in this population. This finding suggests that depending on the content of self-disclosures, a lower amount of self-disclosure was associated with pre-frailty and frailty.

A lower amount of self-disclosure of recent positive events and motivation and strengths in life was associated with frailty. An additional analysis with an interaction term also showed a main effect for self-disclosure, with lower self-disclosure associated with frailty. Socioemotional selectivity theory, a life-span theory of motivation, maintains that as time horizons shrink with age, people typically become increasingly selective and invest more resources in emotionally meaningful goals and activities [37]. Therefore, older adults are assumed to seek positive information about the future and define motivational goals. Apathy is defined as a “lack of motivation” to perform activities such as goal-directed behavior and is associated with the KCL [38]. Individuals who do not engage in positive self-disclosure owing to apathy may not seek information about their motivations and strengths, which may lead to frailty.

Self-disclosures of relationships with family and relatives and experiences of work and social activities were associated with frailty. Social frailty components, such as living alone, visiting friends sometimes, and feeling helpful to friends or family, strongly impact the risk of disability [14]. Social activity differs according to the level of social participation associated with physical frailty [11]. As for life circumstances, older adults may have a smaller social network due to retirement, their own or family members’ illnesses, or the death of acquaintances. Therefore, participants’ limited opportunities for self-disclosure to friends and acquaintances due to their low social participation may have been associated with frailty. Specifically, having psychological resistance to self-disclosure may not be conducive to disclosure [18] as this study’s results indicate an association between self-disclosures of financial status and frailty. As such, in addition to social participation and networks [11,12,13,14], talking about work and social experiences is key to maintaining social connections and a countermeasure against frailty.

The self-disclosure items related to concerns about social interaction, physical health, and cognitive function were not associated with frailty. A previous study observed that negative content, such as a deteriorating physical condition and stress, is disclosed less than positive content, such as beliefs and desires [20]. It was hypothesized that self-disclosure of more negative content due to functional problems meant that more of this content would be associated with frailty. Self-disclosure of this content is important for obtaining social support and preventing the need for care. This study’s results suggest that the frailty group was comparable to the robust group and did not engage in more self-disclosure of concerns about social interactions, physical health, or cognitive functioning. In an additional analysis with interaction terms, there were no main effects for these self-disclosures according to frailty. However, a significant interaction effect was found between self-disclosure and gender when the self-disclosure pertained to concerns about cognitive function. This finding implies that low self-disclosure in men and high self-disclosure in women may predict frailty. The negative ramifications of disclosure, such as feeling vulnerable, uncomfortable, or weak, and being rejected by the person to whom they reveal information, are predicted more strongly among men than women [39]. It can be difficult for men to express concerns about cognitive function, which may be connected to frailty. In such cases, one potential intervention method is establishing an environment in which men can express their concerns after exploring the reasons why self-disclosure is difficult, such as lacking a partner, lacking awareness of cognitive decline, or experiencing personality influences. Conversely, women report more concerns related to subjective cognitive decline [40] and a more frequent incidence of these concerns than men [41]. Interventions aimed at women must address their expressed concerns regarding cognitive decline. Moreover, as part of prevention and rehabilitation services, it is important to provide opportunities for older adults who want to engage in self-disclosure or interact with others but cannot do so.

This study has some limitations. Self-disclosure was measured using a questionnaire, which may not reflect the actual amount of self-disclosure among the participants. Moreover, the content of older adults’ self-disclosures may vary; therefore, identifying the broad scope of actual self-disclosure beyond its content is important to accurately measure it. Furthermore, this study was conducted in a specific region of Japan, and participants were limited to those enrolled in the WHITE study. The proposed questionnaire must still be further tested in other populations, but our initial results may contribute to preventing frailty and improving mental health among community-dwelling older adults.

## Figures and Tables

**Table 1:** Demographic characteristic comparison between the robust and frailty groups.

Variables	Overall (*n* = 237)	Robust (*n* = 117)	Frail (*n* = 120)	Statistics	*p*-Value
Age, years	75.8 ± 5.1	75.3 ± 4.7	76.3 ± 5.4	*t* = 2.611 ^a^	0.104
Gender, women	155 (65.4%)	67 (57.3%)	88 (73.3%)	*χ*^2^ = 6.759 ^c^	0.009 **
Education, years	13.2 ± 2.3	13.5 ± 2.3	13.0 ± 2.4	*t* = 1.492 ^a^	0.137
Medication, *n*/day	2.6 ± 2.7	2.0 ± 2.1	3.2 ± 3.1	*t* = −3.380 ^b^	<0.001 **
Living alone status	66 (27.8%)	28 (23.9%)	38 (31.2%)	*χ*^2^ = 1.764 ^c^	0.184
Working status	44 (27.8%)	27 (23.0%)	17 (14.1%)	*χ*^2^ = 3.111 ^c^	0.078
KCL— Total score	4.9 ± 2.7	1.8 ± 0.9	6.2 ± 2.0	*t* = −21.441 ^b^	<0.001 **

Note. ** *p* < 0.01. ^a^ Student’s *t*-test. ^b^ Welch’s *t*-test. ^c^ *χ*^2^ test.

**Table 2:** Validity of the 13 self-disclosure items.

Self-Disclosure Items	Mean ± SD	G-P	I-T (*ρ*)	WHO-5-J (*β*)	Inter-Item Correlation												
					1	2	3	4	5	6	7	8	9	10	11	12	13
1. Daily life activities	3.3 ± 1.0	1.7 **	0.721 **	0.153 *	—	0.753 **	0.656 **	0.586 **	0.554 **	0.595 **	0.556 **	0.528 **	0.591 **	0.452 **	0.512 **	0.456 **	0.432 **
2. Recent positive events	3.1 ± 1.1	1.8 **	0.773 **	0.261 **		—	0.692 **	0.636 **	0.594 **	0.612 **	0.589 **	0.558 **	0.564 **	0.509 **	0.583 **	0.522 **	0.489 **
3. Motivation and strengths in life	2.8 ± 1.1	1.9 **	0.812 **	0.253 **			—	0.736 **	0.632 **	0.729 **	0.648 **	0.604 **	0.643 **	0.620 **	0.596 **	0.466 **	0.466 **
4. Precious memories	2.8 ± 1.1	1.9 **	0.807 **	0.228 **				—	0.692 **	0.653 **	0.599 **	0.676 **	0.603 **	0.563 **	0.663 **	0.503 **	0.509 **
5. Personality	2.7 ± 1.0	1.8 **	0.816 **	0.221 **					—	0.710 **	0.630 **	0.673 **	0.630 **	0.602 **	0.684 **	0.578 **	0.556 **
6. Wisdom and beliefs	2.8 ± 1.0	1.9 **	0.815 **	0.227 **						—	0.652 **	0.589 **	0.717 **	0.583 **	0.631 **	0.549 **	0.481 **
7. Future outlook	2.8 ± 1.1	2.0 **	0.799 **	0.198 **							—	0.676 **	0.597 **	0.558 **	0.589 **	0.629 **	0.593 **
8. Relationships with family and relatives	2.7 ± 1.1	1.9 **	0.831 **	0.201 **								—	0.652 **	0.693 **	0.698 **	0.636 **	0.625 **
9. Experiences of work and social activities	2.9 ± 1.1	2.0 **	0.777 **	0.198 **									—	0.549 **	0.606 **	0.498 **	0.461 **
10. Financial status	2.2 ± 1.0	1.8 **	0.777 **	0.183 **										—	0.666 **	0.566 **	0.587 **
11. Concerns about social interaction	2.4 ± 1.1	1.9 **	0.828 **	0.072											—	0.671 **	0.625 **
12. Concerns about physical health	2.8 ± 1.1	1.9 **	0.745 **	0.020												—	0.712 **
13. Concerns about cognitive function	2.7 ± 1.2	2.1 **	0.728 **	0.124													—

Note. * *p* < 0.05, ** *p* < 0.01.

**Table 3:** Descriptive statistics on self-disclosure.

Self-Disclosure Items	Robust (*n* = 117)		Frail (*n* = 120)		*U*	*p*-Value
	Mean ± *SD*	Median (*IR*)	Mean ± *SD*	Median (*IR*)		
(a) Recent positive events	3.2 ± 1.1	3.0 (2.5–4.0)	3.0 ± 1.1	3.0 (2.0–4.0)	6516.5	0.319
(b) Motivation and strengths in life	3.0 ± 1.0	3.0 (2.0–4.0)	2.7 ± 1.1	3.0 (2.0–3.0)	5986.5	0.041 *
(c) Personality	2.8 ± 1.0	3.0 (2.0–3.0)	2.7 ± 1.0	3.0 (2.0–3.0)	6645.0	0.457
(d) Future outlook	2.9 ± 1.0	3.0 (2.0–4.0)	2.8 ± 1.1	3.0 (2.0–4.0)	6477.0	0.284
(e) Relationships with family and relatives	2.8 ± 1.0	3.0 (2.0–4.0)	2.6 ± 1.1	3.0 (2.0–3.8)	6592.0	0.401
(f) Experiences of work and social activities	3.0 ± 1.1	3.0 (2.0–4.0)	2.7 ± 1.1	3.0 (2.0–3.8)	5631.0	0.006 **
(g) Financial status	2.3 ± 1.0	2.0 (1.0–3.0)	2.1 ± 1.0	2.0 (1.0–3.0)	6141.0	0.081
(h) Concerns about social interaction	2.5 ± 1.0	2.0 (2.0–3.0)	2.4 ± 1.1	2.0 (1.0–3.0)	6704.5	0.536
(i) Concerns about physical health	2.8 ± 1.0	3.0 (2.0–4.0)	2.9 ± 1.1	3.0 (2.0–4.0)	6869.0	0.766
(j) Concerns about cognitive function	2.6 ± 1.1	3.0 (2.0–3.5)	2.8 ± 1.2	3.0 (2.0–4.0)	6398.0	0.224

Note. * *p* < 0.05, ** *p* < 0.01. Mann–Whitney U test.

**Table 4:** Odds ratios and 95% confidence intervals for frailty according to self-disclosure to friends and acquaintances.

Self-Disclosure Items	Robust (*n* = 117)	Frail (*n* = 120)		
	Reference	Model 1	Model 2	Model 3
(a) Recent positive events	1	0.87 (0.69–1.10)	0.79 (0.61–1.02)	0.74 (0.57–0.97) *
(b) Motivation and strengths in life	1	0.76 (0.59–0.97) *	0.73 (0.56–0.94) *	0.70 (0.53–0.92) *
(c) Personality	1	0.91 (0.71–1.16)	0.82 (0.62–1.07)	0.77 (0.58–1.02)
(d) Future outlook	1	0.87 (0.69–1.10)	0.80 (0.62–1.04)	0.78 (0.60–1.02)
(e) Relationships with family and relatives	1	0.89 (0.70–1.13)	0.80 (0.61–1.03)	0.75 (0.57–0.99) *
(f) Experiences of work and social activities	1	0.73 (0.57–0.92) **	0.69 (0.54–0.89) **	0.66 (0.51–0.87) **
(g) Financial status	1	0.80 (0.62–1.03)	0.77 (0.59–1.01)	0.73 (0.55–0.97) *
(h) Concerns about social interaction	1	0.93 (0.74–1.18)	0.88 (0.68–1.13)	0.81 (0.62–1.07)
(i) Concerns about physical health	1	1.02 (0.80–1.30)	0.98 (0.76–1.27)	0.94 (0.72–1.22)
(j) Concerns about cognitive function	1	1.17 (0.94–1.46)	1.12 (0.88–1.41)	1.10 (0.86–1.40)

Note. * *p* < 0.05, ** *p* < 0.01. Logistic regression analysis. Model 1, a crude model; Model 2, with age and gender adjusted to Model 1; Model 3, with years of education, medication status, living alone status, and work status adjusted to Model 2.

**Table 5:** Odds ratios and 95% confidence intervals for frailty according to self-disclosure, including self-disclosure and gender interaction terms.

Self-Disclosure Items	Men		Women		Odds Ratios and 95% Confidence Intervals for Frail		
	Robust (*n* = 50)	Frail (*n* = 32)	Robust (*n* = 67)	Frail (*n* = 88)	Self- Disclosure	Gender	Self-Disclosure × Gender
(a) Recent positive events	2.8 ± 1.1	2.5 ± 1.3	3.5 ± 0.9	3.2 ± 1.0	0.70 (0.46–1.06)	3.10 (1.51–6.36) **	1.10 (0.64–1.90)
(b) Motivation and strengths in life	2.9 ± 1.1	2.3 ± 1.2	3.1 ± 0.9	2.9 ± 1.0	0.69 (0.50–0.95) *	2.88 (1.44–5.76) **	1.05 (0.72–1.54)
(c) Personality	2.3 ± 1.0	2.2 ± 0.9	3.1 ± 0.9	2.8 ± 1.0	0.79 (0.48–1.28)	2.99 (1.45–6.16) **	0.96 (0.53–1.75)
(d) Future outlook	2.6 ± 1.2	2.2 ± 1.1	3.1 ± 0.9	3.0 ± 1.1	0.63 (0.40–0.98) *	3.25 (1.55–6.79) **	1.43 (0.82–2.51)
(e) Relationships with family and relatives	2.4 ± 1.0	2.0 ± 1.1	3.0 ± 1.0	2.8 ± 1.0	0.69 (0.43–1.11)	3.24 (1.55–6.78) **	1.14 (0.64–2.02)
(f) Experiences of work and social activities	2.8 ± 1.1	2.4 ± 1.1	3.2 ± 1.0	2.8 ± 1.1	0.66 (0.42–1.02)	3.03 (1.49–6.13) **	1.02 (0.59–1.78)
(g) Financial status	2.1 ± 1.1	1.8 ± 0.9	2.5 ± 0.9	2.2 ± 1.0	0.71 (0.43–1.17)	2.82 (1.40–5.66) **	1.04 (0.57–1.91)
(h) Concerns about social interaction	2.1 ± 1.0	1.9 ± 1.0	2.7 ± 1.0	2.6 ± 1.1	0.71 (0.43–1.16)	3.00 (1.45–6.21) **	1.22 (0.68–2.18)
(i) Concerns about physical health	2.6 ± 1.0	2.4 ± 1.1	3.0 ± 1.0	3.0 ± 1.1	0.77 (0.48–1.24)	2.76 (1.36–5.62) **	1.33 (0.75–2.36)
(j) Concerns about cognitive function	2.3 ± 1.0	2.0 ± 1.1	2.7 ± 1.2	3.0 ± 1.1	0.64 (0.40–1.03)	3.02 (1.44–6.32) **	2.09 (1.19–3.66) *

Note. * *p* < 0.05, ** *p* < 0.01. Logistic regression analysis adjusted for age, years of education, number of medications, living alone status, and work status.

## Data Availability

The data presented in this study are available upon request from the corresponding author upon reasonable request. The datasets are not publicly available because they contain information that may infringe on the privacy of the study participants.
